# Glancing at the role of *Toxoplasma gondii* in neuro-oncology: from risk factor to therapeutic target

**DOI:** 10.1186/s13027-026-00747-6

**Published:** 2026-03-26

**Authors:** Maha M. Eissa, Noor Al-Huda F. Ali, Nahla El Skhawy

**Affiliations:** 1 Department of Medical Parasitology, Faculty of Medicine, Alexandria University, Alexandria, Egypt; 2 Joint MB ChB/ MBBCH Programme, Faculty of Medicine, Alexandria University-Manchester University, Alexandria, Egypt

**Keywords:** Antineoplastic, Biological therapy, Brain tumors, Cancer immunotherapy, *Toxoplasma gondii*

## Abstract

**Background:**

Cancer is a leading cause of morbidity and mortality, with brain tumors posing significant challenges due to their site and complexity. Therefore, exploring tumor pathogenesis and the discovery of novel therapeutic agents have drawn attention to the role of infectious agents. *Toxoplasma gondii* is a highly prevalent protozoan parasite that establishes a chronic latent infection in neural tissues. Several epidemiological studies have linked infection with *Toxoplasma gondii* to a higher risk of developing cancer, particularly brain tumors. However, research findings are controversial, with some reports suggesting no clear link, and others even proposing that *Toxoplasma gondii* infection could ignite the immune system, consequently fighting brain cancer.

**Main body:**

In this review, we screened several major scientific databases for studies published up to July 2025. We included human and experimental studies that explored the potential oncogenic and oncosuppressive effects of *Toxoplasma gondii* in brain tumors. While some epidemiological studies suggest that *Toxoplasma gondii* infection is a risk factor, others have pointed out that the incidence of this parasitic infection may be due to the opportunistic nature of the parasite, leading to reactivation of latent infection or acquiring a new infection in immunosuppressed cancer patients. Additionally, pre-clinical studies have shown that *Toxoplasma gondii* could boost the immune cells and modify the tumor microenvironment to fight brain tumors.

**Conclusion:**

This review emphasizes the potential role of *Toxoplasma gondii* as a valuable resource that warrants further exploration for its promising anti-neoplastic activity against brain cancer, as well as other types of cancer.

## Introduction

Cancer is a leading cause of death globally, surpassing stroke and heart disease in many countries [1]. The incidence and mortality rates of cancer are on the rise worldwide due to factors like aging, population growth, and changes in risk factors [2, 3]. In 2020, there were 19.3 million new cancer cases and nearly 10 million cancer-related deaths. By 2040, an estimated 28.4 million new cancer cases are projected, representing a 47% increase from 2020. Efforts to control cancer must be globally escalated to address its diverse causes [1].

The top 10 cancer types, including breast, lung, colorectal, prostate, and stomach cancers, account for a significant portion of new cases and deaths globally. Lung cancer is the most deadly form of cancer, followed by colorectal, liver, stomach, and breast cancers [4]. Brain and CNS tumors account for 1.6% of all tumors, with an annual incidence of 9 per 100,000 for primary tumors and 8.3 per 100,000 for metastatic tumors. Brain tumors, while rare compared to other types of cancer, pose significant treatment challenges due to their sensitive location and the potential risk of damaging essential neural structures during treatment [5].

Brain and CNS tumors can be classified based on histopathology or location. Primary tumors originate in the brain or CNS tissues, with over 100 distinct types known as ‘histopathologies’, each with unique clinical presentations, treatments, and outcomes. In contrast, metastatic tumors spread to various brain locations and represent 15%-25% of all brain tumors [5, 6]. Gliomas, including astrocytomas, oligodendrogliomas, and ependymomas, are the most common primary brain tumors and represent 40%-55% of all brain tumors [7]. Glioblastoma is the most common malignant tumor, accounting for 14.2% of all tumors and 50.1% of malignant tumors, with a higher prevalence in males [5–7]. Other types of brain tumors include meningiomas, schwannomas, craniopharyngiomas, germ cell tumors, pituitary adenomas, and pineal region tumors. Meningioma is the most common non-malignant tumor, accounting for 39.7% of all tumors and 55.4% of all non-malignant tumors, with a higher prevalence in females [5, 7].

Tumor location can impact symptoms, with dorsolateral tumors affecting organization and planning, orbitofrontal tumors causing disinhibition, and medial frontal tumors leading to apathy. Frontal tumors may result in personality changes, while diencephalic and pituitary lesions can cause vegetative symptoms [8–10]. Between 2015 and 2019, there were 84,264 deaths from malignant brain and CNS tumors, resulting in an average annual mortality rate of 4.41 per 100,000 population and an average of 16,853 deaths per year from these tumors [5].

Early diagnosis of brain tumors can be difficult because symptoms often start gradually and are vague and nonspecific. Radiological imaging, such as CT and MRI scans, is commonly used for diagnosis, with MRI being more precise, especially in complex cases [11]. Histological examination of tumor tissue is the gold standard for grading tumors and planning treatment [12]. Treatment usually involves surgery, chemotherapy, and radiation, but these methods can also damage nearby healthy tissues unintentionally [13].

Risk factors for brain and CNS tumors include heritable genetic factors, allergic/atopic diseases, and ionizing radiation exposure [7]. Nevertheless, the specific reasons behind these cancers remain unknown. Around 20% of cancer cases globally are caused by infectious agents such as viruses, bacteria, and parasites, with higher rates in low-income countries [14–16]. These agents can disrupt the host cell’s genetic processes, like DNA repair and cell cycle, resulting in chronic inflammation and immune system dysfunction [17].

Infectious pathogens are significant and modifiable factors that contribute to the development of cancer. Many cancer-causing infections can be prevented, with existing tools for prevention and intervention. High-risk alpha human papillomaviruses (HPV), hepatitis B (HBV) and C (HCV) viruses, and *Helicobacter pylori* bacterium are responsible for over 90% of infection-related cancers globally [16]. Additionally, human T-cell lymphotropic virus 1 (HTLV-1), Epstein–Barr virus (EBV), Kaposi sarcoma-associated herpesvirus (HHV-8), as well as the *Opisthorchis viverrini*, *Clonorchis sinensis*, and *Schistosoma haematobium* helminths have been classified as class 1 carcinogens by the International Agency for Research on Cancer. These oncogenic pathogens share common abilities to promote cell survival and transformation through chronic insults, genetic and epigenetic alterations, deregulated metabolic pathways, and immune evasion [18].

The burden of pathogen-related cancers is expected to increase as new oncogenic microorganisms are identified. Notably, HPV infection plays a significant role in oropharyngeal cancers, highlighting the clinical relevance of this association [19]. The interaction between viruses and environmental risk factors, such as cutaneous HPV types, accompanied by ultraviolet radiation [20] or HBV with aflatoxin [21], is an emerging issue in cancer development. Recent research has also highlighted the impact of microbiota on cancer susceptibility, neoplastic progression, co-infections with carcinogenic agents, and response to therapy [22, 23].

While some parasitic infections, such as *Schistosoma haematobium*, *Opisthorchis viverrini*, and *Clonorchis sinensis*, are well-established promoters of human cancer, studies on other parasitic infections remain inconclusive and require further validation. For example, *Plasmodium falciparum* is associated with Burkitt lymphoma despite its non-carcinogenic role [24].

*Toxoplasma gondii* (*T. gondii*), an intracellular protozoan parasite, is prevalent in humans worldwide, with nearly one-third of the population estimated to be infected with a wide geographic variability [25, 26]. It has an affinity for neural tissue and can form cysts in the brain during chronic infection. *Toxoplasma gondii* cysts can persist for the host’s lifetime without triggering an immune attack [27]. The parasite can alter the microenvironment of infected hosts and has been linked to brain tumor growth [28]. Yet, the genuine relationship between *T. gondii* and cancer has been a controversial point and a focus of multiple research studies, both epidemiologically and experimentally [29]. Epidemiological studies have indicated a potential link between *Toxoplasma* infection and an increased risk of brain cancers [30–36], with growing interest in *Toxoplasma*’s role in brain tumor development, although not all researchers agree [30, 37–39]. On the other hand, a plethora of experimental work has demonstrated that *Toxoplasma* may have a protective effect against various types of cancer, including brain tumors, based on findings in mouse models [40].

This review aims to comprehensively analyze the existing data on the impact of *Toxoplasma* on brain tumors. It will also discuss the mechanisms through which *Toxoplasma* may interact with brain tumor cells and the implications of these interactions for the development or regression of brain tumors. Overall, this review will provide valuable insights into the complex relationship between *Toxoplasma* infection and brain tumors, shedding light on potential avenues for future research and treatment.

## Database search

To ensure a comprehensive review of the literature, we conducted searches on various electronic databases such as PubMed, ScienceDirect, Scopus, the Egyptian Knowledge Bank, and Google Scholar. The search aimed to gather relevant epidemiological, human and pre-clinical research that investigates the role of *T. gondii* in neuro-oncology, including mechanistic studies. Various combinations of search terms were used, including “*Toxoplasma gondii*, Toxoplasmosis, parasites, protozoa, helminths”, and “cancer, brain tumors, CNS tumors, glioma, glioblastoma, meningioma, neuro-oncology, cancer inducer, cancer suppressor, cancer immunotherapy”. Studies were selected based on relevance to the review topic rather than strict methodological hierarchy. The selection criteria included peer-reviewed original research, reviews, human studies, and pre-clinical studies published up to July 2025. Both human and experimental research were included to cover all studies addressing the complex relationship and impact of *T. gondii* on brain and CNS tumors. The articles’ content was thoroughly examined, as well as checked their references for additional relevant studies. Non-peer-reviewed publications, conference papers and case reports lacking sufficient data were excluded. Given the heterogeneity of the studies, a qualitative narrative synthesis was performed to organize the findings, focusing on the role of *T. gondii* in neuro-oncology as a potential oncogenic pathogen, an opportunistic infection, and a promising tool for cancer immunotherapy.

## Results

**Fig. 1:**
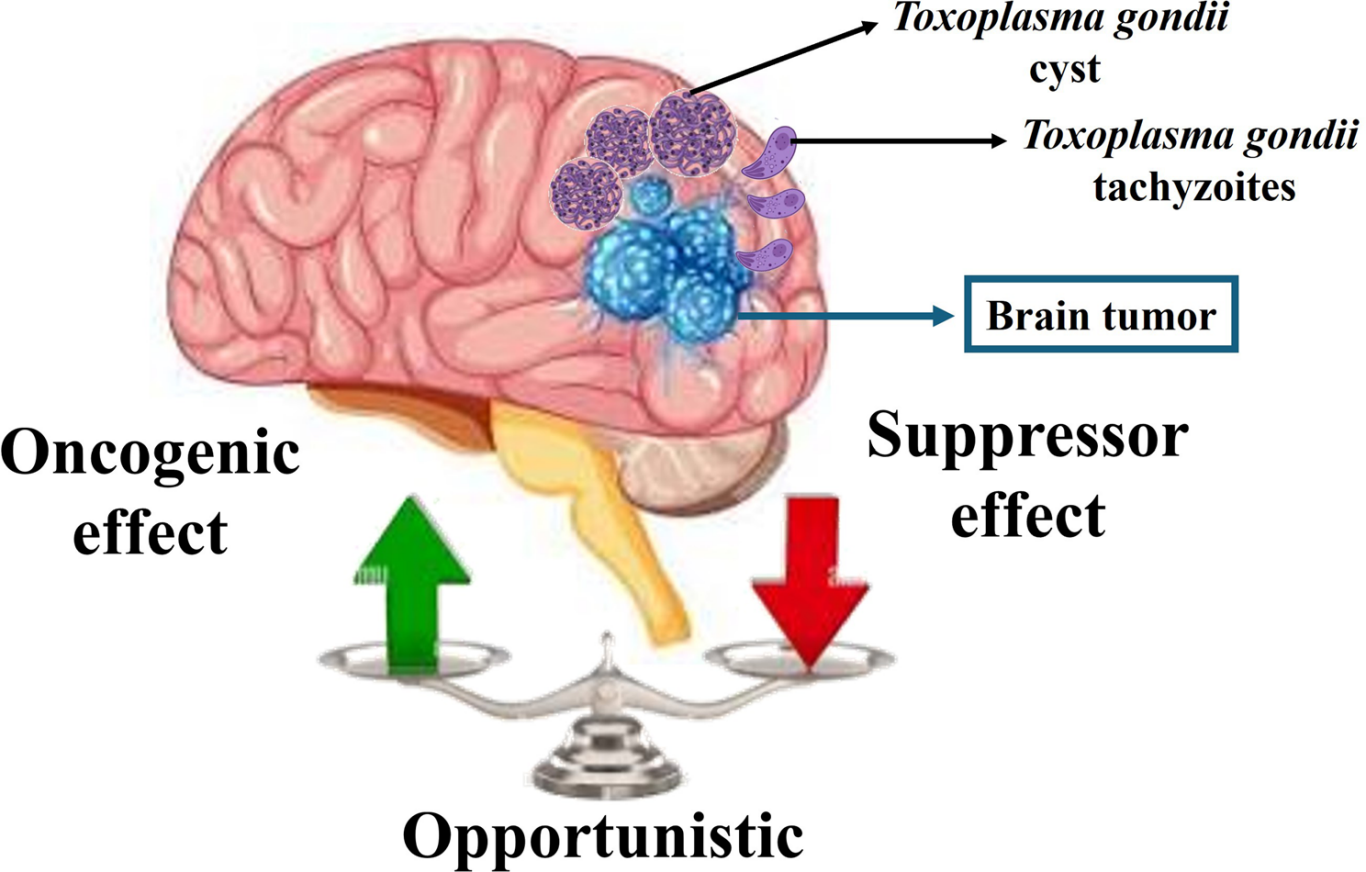
Schematic diagram illustrating the dualistic role of *T.gondii* in neuro-oncology

A literature review revealed that *T. gondii* has a dualistic role in the development of brain tumors (Fig. 1). Some human epidemiological studies suggest that *T. gondii* may be a risk factor for brain tumors. In contrast, others clarified that its high prevalence in immunosuppressed brain tumor patients could be due to its opportunistic nature. Interestingly, this review revealed an intriguing finding about *T. gondii* ’s anti-neoplastic properties against brain tumors in pre-clinical studies.

### *Toxoplasma gondii* as a potential risk factor for brain tumor development

Several epidemiological studies have suggested a potential oncogenic role of *T. gondii* in the development of brain tumors. The initial study, published in 1967, found that 56% of individuals with brain tumors tested positive for toxoplasmosis using a dye test, compared to 41% of controls. The highest rate of positivity was among gliomas specifically, astrocytomas [41]. In 1993, further research was conducted in Adelaide, South Australia, as part of Australian population-based case-control studies. The study aimed to investigate the potential link between previous infection with *T. gondii* and brain tumor development. The Enzyme-linked Immunosorbent Assay (ELISA) technique was used to detect IgG antibodies to *T. gondii*. The results showed a statistically significant increased risk of meningioma in individuals who tested positive for *Toxoplasma* antibody, indicating a possible association with meningioma [30]. Subsequent epidemiological studies across 37 countries and 22 regions in France have suggested a potential link between *T. gondii* infection and brain tumors. Regional mortality rates from brain cancer were found to positively correlate with the local seroprevalence of *T. gondii*, especially among men [31, 32]. Similar findings were observed in studies conducted in China, Korea, and Egypt using different detection methods such as ELISA and electrochemiluminescence immunoassay [36, 42, 43]. Two prospective cohort studies also indicated a higher risk of glioma among individuals infected with *T. gondii*, particularly those with high antibody titers to specific *T. gondii* SAG-1aA antigen [44]. A meta-analysis of multiple studies further supported an increased risk of brain tumor occurrence, particularly gliomas and meningiomas, associated with *T. gondii* infection [33]. Recent studies have continued to demonstrate a higher seroprevalence of *T. gondii* infection in brain tumor patients compared to healthy controls, as assessed by the ELISA technique [34, 35]. The study by Asgari et al., in 2023, observed a specific association between seropositivity for *T. gondii* infection and different types of brain tumors, with ependymoma showing the highest correlation, followed by glioblastoma, pituitary adenoma, astrocytoma, schwannoma, and meningioma in descending order. Furthermore, they demonstrated that the location of the brain tumor also played a role, as patients with tumors in the frontal lobe and sella region had a higher likelihood of *T. gondii* infection [34]. While the study of Hamouda et al., in 2024, supported the hypothesis that *T. gondii* infection may be a risk factor for childhood brain tumors by demonstrating a significantly higher seroprevalence of *T. gondii* infection in brain tumor cases compared to healthy controls, with glioma cases showing 100% seroprevalence across other different subtypes of brain tumors that showed variable rates [35].

### Opportunistic nature and prevalence of *Toxoplasma gondii* in brain tumor patients

A study by Ryan et al. in 1993 found no association between *T. gondii* infection and the development of glioma. They observed no difference in anti-*Toxoplasma* antibody positivity between glioma patients and controls (35% in glioma patients versus 33% in controls) using an ELISA test [30]. Benson et al. in 2012 also found no increased risk of brain cancer in women owning cats compared to non-cat owners [45]. Additionally, a case of a 12-year-old boy showed the presence of the *T. gondii* parasite in the CSF after immunosuppressive therapy following astrocytoma removal [46]. Other epidemiological studies suggest that toxoplasmosis can be an opportunistic infection in immunosuppressed individuals, including cancer patients, especially after undergoing chemotherapy [47–57]. A summary of the clinical studies presenting the relation between *T.gondii* and neuro-oncology is shown in Table 1.

**Table 1:** A Summary of clinical studies investigating the role of *T. gondii* in neuro-oncology

Study type	Country	Brain tumor type	Method of detection of T.gondii infection	Results	Conclusion	Reference
Clinical study (a retrospective study)	Saint Paul, Minnesota, United States	Central Nervous System Neoplasms	Sabin-Feldman dye test	*T. gondii* positivity was found in 56% of brain tumor patients, with the highest rate in glioma patients, particularly astrocytomas, compared to 41% of controls	A positive association of *T.gondii* infection and CNS tumors (glioma, astrocytomas	[41]
Clinical studies (two case-control studies )	Adelaide, Australia Melbourne, Australia	• Glioma and meningioma • Glioma	Enzyme-linked Immunosorbent Assay (IgG)	• In both studies no differences in seropositivity between glioma patients and controls • Higher risk of meningioma in patients positive for *T.gondii* IgG (47% test-positive versus 31% in controls)	No evidence that *T.gondii* seropositivity is a risk factor for glioma, but it may be associated with meningioma	[30]
Database [Ecological study - A medical geography based on the national data of *T gondii* seroprevalence (cross-sectional studies) and incidence of brain cancers, from population-based registry (the International Agency for Research on Cancer)]	Several countries such as France, Brazil, the UK, the USA, Mexico, Turkey, South Korea, China, India, among others)	Brain cancers	Various serological tests, depending on the resources available in each country (for the detection of *T.gondii* IgG antibodies)	Higher national seroprevalence of *T. gondii* was linked with a 1.8-fold increase in adult brain cancers risk	The findings are correlational, and further research is needed	[31]
Database (National data for *T.gondii* seroprevelance and mortality rates from brain cancer )	France (in 22 administrative regions)	Brain cancer mortality	Database (Serological screening (ELISA and IFA tests for detection of *T.gondii* IgG antibodies)	Brain cancer mortality rates correlate positively with higher *T.gondii* seroprevelances, particularly in men above 55 years old	Further individual-level analyses are needed to confirm causality	[32]
Clinical study (case control study)	Korea	Brain tumors	Serological test (ELISA)	Brain tumor patients had a significantly higher rate of *T.gondii* infection (18.3%) compared to healthy controls (8.6%), with a higher infection rate in younger brain tumor patients compared to age-matched controls	The study supports the link between *T.gondii* and brain tumor incidence	[36]
Clinical study (a case-control study)	China	Cancer patients, including brain cancer patients	Serological test (ELISA)	High *T.gondii* seroprevelance in brain cancer patients (42.31%)	*T. gondii* is a serious infection in cancer patients, and measures to prevent and control infection in those patients are important	[42]
Clinical study (a case-control study)	Egypt	Different types of human tumors including brain tumors	Electrochemiluminescence immunoassay	A positive correlation between *T.gondii* seropositivity and brain tumors in 69.23% of patients	*T.gondii* could be a potential risk factor for brain tumors	[43]
Two prospective cohort studies (a nested case-control study design)	The United States	Glioma	ELISA (IgG)	High antibody titers to *T. gondii* SAG-1aA antigen linked to increased glioma risk	More research is needed to confirm *T.gondii’s* potential role in glioma	[44]
Clinical study (a case-control study)	Southern Iran	Brain tumors	ELISA (IgG)	• Seroprevalence of anti-Toxoplasma IgG was significantly higher in brain tumors patients (30.6%0 versus healthy controls (12.1%) Infection was correlated with tumor location, with patients with frontal lobe and sella region tumors showing higher seropositivity	The study findings suggested a possible link between *T.gondii* infection and the development of brain tumors	[34]
Clinical study (a case-control study)	Egypt	Childhood brain tumors	ELISA	• Higher *T.gondii* seroprevlance in brain tumor cases (62.5%) compared to healthy controls (38%) • Glioma cases had a 100% • seroprevalance	*T.gondii* infection may increase the risk of childhood brain tumors, highlighting the need for further research	[35]
Database (a large UK prospective cohort to Investigate the link between owning a cat and the risk of brain tumors in middle- aged women.)	The United Kingdom	CNS tumors including glioma and meningioma	Cat ownership survey data	• No association was found for any type of CNS tumor, including glioma or meningioma	Owing a cat is not a high risk factor for brain cancer, however there is a possibility that *T.gondii* infection from other sources could be linked to brain cancer incidence	[45]
Case study	Canada	A child with an astrocytoma	CSF examination for *T.gondii* tachyzoites	• *T.gondii* presence in immunocompromised patients	Opportunistic nature of *T.gondii*	[46]

### *Toxoplasma gondii* as a potential suppressor factor for brain tumor

In contrast to several epidemiological studies suggesting *T. gondii* as a potential risk factor for brain tumor development, a multitude of experimental studies have highlighted the anti-neoplastic properties of this parasite against various types of cancer. Out of a round 50 experimental research studies using *T. gondii* or its derivatives on cancer, only three have specifically focused on brain tumors such as gliomas, medulloblastomas, and ependymoblastoma [40].

The first study conducted by Conley et al. in 1977 demonstrated the suppressive effect of *T. gondii* infection on a C57BL/6J mouse model of ependymoblastoma. The infected mice showed a significant reduction in tumor growth, accompanied by a robust inflammatory response around the tumor with increased necrosis compared to the control mice. Notably, peritoneal macrophages from *Toxoplasma*-infected mice exhibited cytotoxicity to ependymoblastoma cells in vitro, while those from the control mice did not [58].

Subsequent research on gliomas revealed that *Toxoplasma* lysate antigen inhibited the in vitro proliferation and invasion of human glioma cells (U373MG and U87MG), in a dose-dependent manner, leading to glioma cell apoptosis at high concentrations. Treatment of athymic nude mice transplanted with human malignant glioma cells with *Toxoplasma* lysate antigen significantly decreased tumor growth, which was further enhanced when adjuvanted with Quil-A [59].

A recent study by Nguyen et al. in 2022 investigated the impact of *T. gondii* on medulloblastomas. They found that infection with *T. gondii* in a mouse model of medulloblastoma decreased tumor incidence by attracting T cells to the brain tumor microenvironment (TME) and creating a supportive environment for T cells. Additionally, there was an increase in interferon-gamma (IFN-γ) and IFN-γ-driven genes in the tumors, indicating stimulation of T-helper 1 (Th1) immunity in the TME [60]. A summary of pre-clinical studies that showed the suppressor effect of *T.gondii* in neuro-oncology is shown in Table 2.

**Table 2:** A summary of pre-clinical studies investigating the effect of *T.gondii* on different types of brain tumors

Study type	Brain tumor type	Toxoplasma infection/ antigen	Results	Conclusion	Reference
• In vivo • In vitro	• C57BL/6J mouse model of ependymoblastoma • Ependymoblastoma cells	• *T.gondii* infection • peritoneal macrophages from *T.gondii* infected mice	• Significant reduction in tumor growth, necrosis and increased inflammation around the tumor • Cytotoxic effect on ependymoblastoma cells	• The results of both studies demonstrated the anti-neoplastic activity of *T.gondii* against ependymoblastoma	[58]
• In vitro • In vivo	• Human glioma cells (U373MG and U87MG), • Athymic nude mice transplanted with human malignant glioma cells	*T.gondii* lysate antigen	• Inhibited the growth and invasion of glioma cells in a dose-dependent manner, resulting in apoptosis at high concentrations • Tumor growth was significantly reduced	• The results of both studies demonstrated the anti-neoplastic activity of *T.gondii* lysate antigen against human malignant glioma	[59]
In vivo	A mouse model of medulloblastoma	*T.gondii* infection	Reduced tumor incidence, modified TME, and stimulated T-helper 1 immunity in TME	The results demonstrated the anti-neoplastic activity of *T.gondii* against medulloblastoma	[60]

## Discussion

Research has primarily focused on the link between parasites, particularly worms, and cancer. However, some protozoa, like *T. gondii*, have also been associated with an increased risk of brain cancer. *Toxoplasma gondii* is a prevalent protozoan parasite that affects a significant portion of the global human population, ranging from 30 to 65% based on the geographic distribution [49]. However, the association of toxoplasmosis with brain tumors is not the only one reported in clinical and epidemiological studies conducted worldwide. Other studies have also suggested a possible link between *T. gondii* seroprevalence and other malignancies, with varying seroprevalence rates. High anti-*T. gondii* antibodies were demonstrated in patients with leukemia, lymphoma [61], lung [62], colon [63, 64], breast [47, 64–66], stomach, hepatocellular carcinomas, oral [67], and bone cancers [42, 43, 68–71] across different countries such as Egypt [43, 49, 69], Turkey [48], Iraq [66, 72], Iran [47, 61, 65, 68], China [63, 67, 73], and England [62]. *Toxoplasma gondii* was also suggested to be involved in raising the risk of intraocular lymphoma [74].

However, the exact mechanism of *Toxoplasma*-induced brain cancer is not fully understood. *Toxoplasma gondii* can reside in the brain for a long time, and the development of brain cancer typically takes decades. *Toxoplasma gondii* is assumed to behave like other intracellular pathogens, promoting cancer via a prolonged state of inflammation, accumulating oncogenic mutations [43, 75], and some data concluding upregulation of vascular endothelial growth factor (VEGF) expression upon *Toxoplasma* infection, mainly in the neural tissue [76, 77]. The parasite inhibits cell self-destruction and induces mild inflammation, both of which are hallmarks of cancer.

Microarray analysis has revealed that *T. gondii* invasion can affect over 1,000 host cell genes, impacting functions such as apoptosis, metabolism, inflammation, and cell growth [78–80]. Research has shown that *Toxoplasma* can influence the expression of host miRNAs, which play a crucial role in the growth and spread of brain cancer [81–83]. *Toxoplasma gondii* can modify the expression of host microRNAs in glial cells, leading to increased miR-132 expression [84, 85]. This miRNA is known to downregulate genes like *APAF1* and *KRAS* [85], which are essential for apoptosis and subsequently cancer development [86, 87].

However, despite the large number of epidemiological studies demonstrating the strong association between *T. gondii* seroprevalence and brain tumors, as well as other tumors, conclusive evidence linking *T. gondii* to the direct causation of these tumors is still lacking. It is crucial to validate these findings with well-designed cohort studies that rigorously control for confounding factors.

Conversely, lower prevalences of anti-*T. gondii* antibodies have been reported in China, Iran, Saudi Arabia, and Jordan, particularly in patients with intracranial malignancies, lymphoma, hepatocellular carcinoma, and breast cancers [49, 55]. Additionally, some studies have shown the absence of anti-*T. gondii* IgM antibodies in pediatric hematologic malignancies [88, 89]. Furthermore, a recent study reported that *T. gondii* is highly prevalent in cancer patients undergoing chemotherapy as well as in healthy controls, with the highest serologic combination being “IgM-, IgG + ” in both groups [49]. Similarly, another study in Iran demonstrated that the seroprevalence of toxoplasmosis in patients with adenocarcinoma, breast cancer, lymphoma, prostate cancer, ovarian cancer, squamous cell carcinoma, multiple myeloma, and pancreatic cancer was the same as that of the healthy group [90]. Additionally, in cancer patients who were positive for *Toxopla*sma IgG, where *Toxoplasma* DNA was detected in fixed tissues of breast cancer, no association was concluded between breast cancer and toxoplasmosis [65].

The rising incidence of cancer, which induces an immunosuppressive status, has attracted significant attention to the probability of acquiring opportunistic infections such as *T. gondii*, *Pneumocystis*, and *Cryptosporidium*. *Toxoplasma gondii* is now recognized as a major opportunistic pathogen in immunocompromised patients [49]. The high prevalence of anti-*T.gondii* antibodies in cancer patients, especially those undergoing chemotherapy, suggest a correlation between *T. gondii* and cancer. However, this does not imply that the parasite causes cancer. The presence of *T.gondii* IgG antibodies may indicate past exposure rather than active infection. Additionally, the immunocompromised status of cancer patients may lead to the reactivation of latent infections or increased susceptibility to new infections, rather than the parasite being an oncogenic agent, raising concerns about reverse causality. Many studies have not adequately controlled for confounding factors, such as variations in environmental factors that facilitate infection transmission, socio-demographic characteristics, dietary habits, genetic susceptibility, and immune status among the studied population. These variations may justify the disparity in prevalence rates in various epidemiological studies across different geographical regions globally. These challenges make it difficult to definitively determine a causal relationship between *T.gondii* and brain tumors based solely on serological data. Therefore, the potential association between *T.gondii* seropositivity in cancer patients should be cautiously analyzed.

In 2017, a global meta-analysis concluded that immunocompromised individuals are more likely to be infected with *T. gondii* [56]. This underscores the importance of including toxoplasmosis screening in their routine medical assessments. Regular evaluation for *Toxoplasma* is essential for these patients, and it is crucial to implement appropriate prevention and control strategies for this at-risk population [48, 53, 56]. Thus, human epidemiological studies investigating the relationship between *Toxoplasma* and brain cancer have yielded inconclusive results.

While some studies have found a high seroprevalence of anti-*Toxoplasma* antibodies in brain cancer patients, it remains unclear whether this association is causal, coincidental, or part of opportunistic infection. Additionally, the lower incidence of cancer in developing countries where parasitic infections are common may indicate a different relationship. Some epidemiological studies have reported that cancer patients infected with certain parasites have better survival rates than non-infected patients [91, 92]. An epidemiological study even suggested that individuals with low levels of anti-*Toxoplasma* antibodies may have a reduced risk of cancer, indicating a potential role of asymptomatic *T. gondii* infection in enhancing anti-tumor immunity [38]. Thus, we needed to elaborate on the anti-neoplastic potential of *T. gondii* against brain tumors. Pre-clinical studies have shown that infection with *T. gondii* or the use of its lysate antigen exhibited potent antineoplastic activity against brain tumors such as gliomas, medulloblastomas, and ependymoblastoma. However, this anti-neoplastic activity against brain tumors is not exclusive to *T. gondii*. Other parasites, such as *Trichinella spiralis*, *Trichomonas vaginalis*, and *Plasmodium species*, have been investigated and also demonstrated similar anti-neoplastic activity and significantly reduced glioma size in murine models [93–96].

**Fig. 2:**
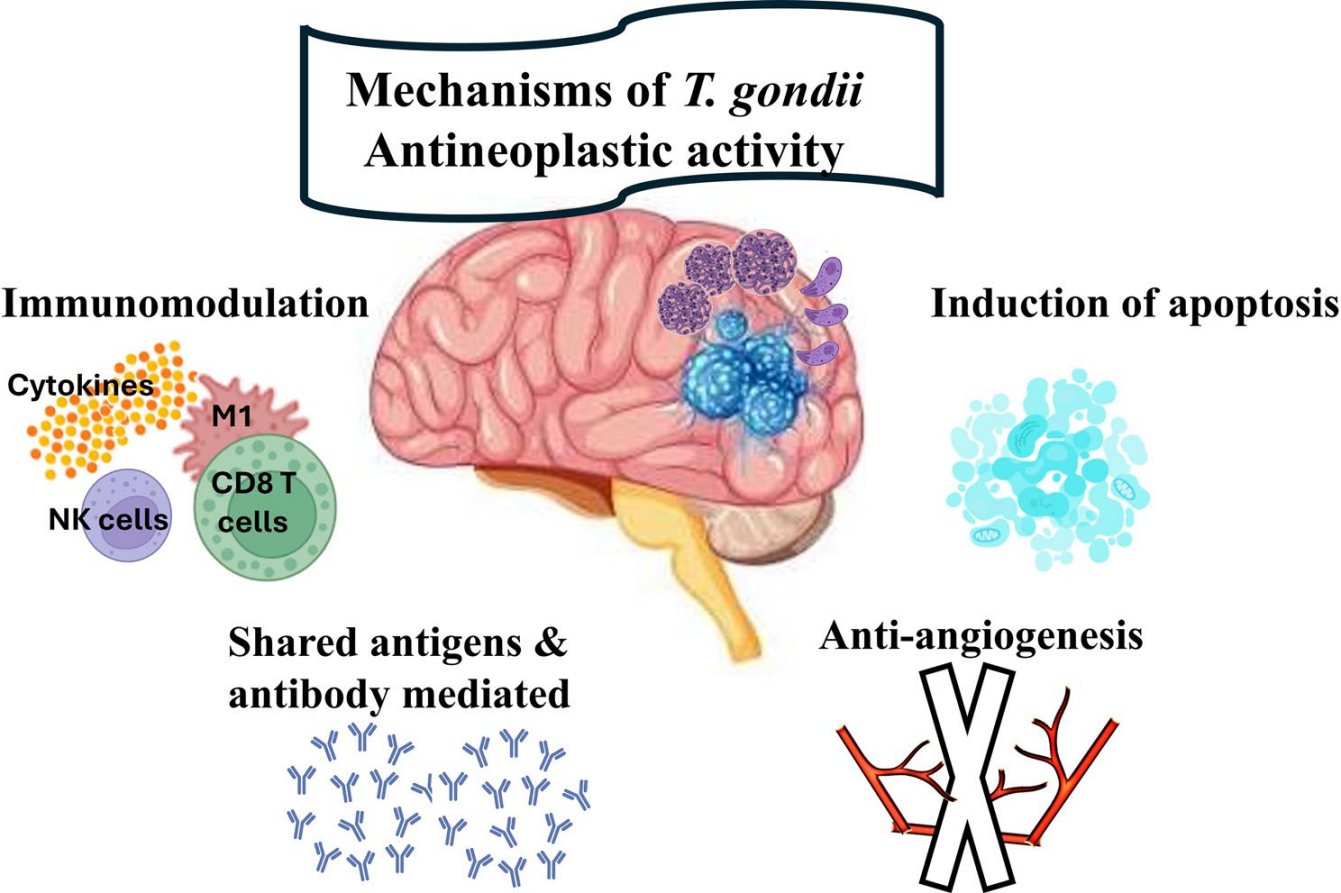
Schematic diagram illustrating the different mechanisms of *T.gondii* antineoplastic activity

*Toxoplasma gondii* has been demonstrated to suppress tumor growth in various murine cancer models through various mechanisms such as immunomodulation, induction of apoptosis, inhibition of angiogenesis, and molecular mimicry with cancer cells (Fig. 2). *Toxoplasma gondii* triggers strong Th1 immune responses [97]. It can convert natural killer (NK) cells into innate lymphoid-like cells (ILC1-like cells) within the TME, leading to the dissemination of cells with immune memory capabilities in the circulation and sustained IFN-γ production [98]. *Toxoplasma gondii* is a potent stimulator of interleukin-12 (IL-12) production, which activates IL-12 expression via the host’s myeloid differentiation factor 88 (MyD88) signalling pathway. IL-12, a multifunctional cytokine, primarily targets NK cells, CD4^+^ and CD8^+^ T lymphocytes, inducing a Th1 cell immune response and inhibiting angiogenesis. Ultimately, CD8^+^ T cells, NK cells, and macrophages are recruited to the tumor tissue to eliminate tumor cells by releasing IL-12 and IFN-γ [99]. Modulation of the TME is a key mechanism of *T. gondii-*based cancer therapy. Studies have shown that immunization with various *T. gondii* as well as other parasites and their derivatives enhances the tumor-inhibitory state by increasing cytotoxic CD8^+^ T cells, NK cells, IFN-γ production, and depleting T regulatory (Treg) cells [100–103]. *Toxoplasma gondii* secretes specialized proteins with immunomodulatory effects, impacting host cell signaling and transcriptional responses. GRA18 promotes anti-inflammatory reactions while ROP16 and ROP38 inhibit specific immune pathways such as the STAT-3/6 pathways, NF-κB pathway, and IL-12 production, respectively [104]. GRA6Nt triggers specific immunity against cancer [105] and GRA15II modulates cytokine profiles [106]. Additionally, profilin-like protein from *T. gondii* acts as a Toll-like receptor agonist, increasing the expression of markers on antigen-presenting cells and cytokines such as IFN-γ and IL-12 [107, 108]. *Toxoplasma gondii* also exhibits an anti-tumor effect by promoting apoptosis in cancer cells, leading to suppressed tumor growth. Surprisingly, *T. gondii* has a dual role in apoptosis, affecting both pro-apoptotic and anti-apoptotic pathways in cells [109]. This involves modulation of mitochondrial pathways, suppression of pro-survival signaling, and activation of caspases in cancer cells. Hence, the parasite remodels the TME, reduces immunosuppression, and enhances antitumor immunity [109, 110]. In contrast, the parasite blocks apoptosis in immune cells like macrophages and dendritic cells, promoting their survival and evading immune clearance, leading to continuous proinflammatory responses and enhanced antitumor immune surveillance. *Toxoplasma gondii* directly inhibits caspase-3 activation and reduces interaction with poly (ADP-ribose) polymerase, which is crucial for preventing excessive cell death, helping preserve the survival of host cells.

This paradoxical induction of apoptosis in cancer cells while suppressing it in immune cells makes it an intriguing candidate for engineered immunotherapies for tumor suppression while maintaining immune function, underscoring the importance of tightly regulating its pathogenic capabilities [109, 110].

*Toxoplasma* is still able to elicit antineoplastic activity through its antiangiogenic capacity, thereby promoting significant hypoxia [97]. Deprivation of the tumor from the blood supply is a strategic approach that has attracted oncologists to trigger tumor cell death [111]. Neoangiogenesis is a crucial factor in tumor growth and metastasis mediated through VEGF. Failure to achieve this factor results in tumor cell death. Through a direct strategy, *T. gondii* suppresses vascular development within tumor tissue via the suppression of VEGF. Interestingly, via an indirect mechanism, VEGF has an immune-suppressive activity by inhibiting the differentiation and proliferation of immune cells (macrophages, monocytes, and CD8 + T cells) while inducing Treg cell proliferation. Consequently, interfering with angiogenesis unleashes the immune system [112]. Thus, targeting angiogenesis is a dual cancer attack strategy by inhibiting VEGF coupled with preventing immune suppression [100, 101]. Several preclinical animal studies in immunocompetent [100, 113, 114] and immunosuppressed models [97] have demonstrated the antiangiogenic effects of *Toxoplasma* and its antigens, leading to the inhibition of tumor growth in mouse cancer models.

Additionally, shared antigens between *Toxoplasma* and cancer cells could be another mechanism for its anti-neoplastic activity [115, 116]. Flow cytometry analysis revealed that both human and rabbit anti-*Toxoplasma* antibodies can bind to breast cancer cell lines (MCF7 and 4T1), indicating the presence of shared epitopes between *T. gondii* and breast cancer cells [117, 118]. Another study confirmed the existence of specific shared epitopes between *T. gondii* and cancer cells through flow cytometry analysis, demonstrating a specific and strong interaction of anti-*T. gondii* antibodies with melanoma cells, but not with anti-*Trichomonas vaginalis* or anti-hydatid cyst fluid and protoscolices antigens, suggesting potential common antigens between melanoma and *Toxoplasma* but not with *Trichomonas vaginalis* or *Echinococcus granulosus* [117]. Furthermore, immunoblotting revealed four cross-reactive bands at molecular weights of approximately 60, 26, 22, and 12.5 kDa between *T. gondii* antigen and Ehrlich carcinoma, a murine mammary carcinoma, suggesting shared antigens between Ehrlich carcinoma and *T. gondii*, supporting the theory of molecular mimicry in parasites’ antineoplastic activity [100].

In human cancer cells, mucin-type O-glycan structures are considered among the highly specific cancer-associated structures [119]. Interestingly, research reports have shown that post-translational N-glycosylation of *T. gondii* tachyzoites is common, and *Toxoplasma* produces numerous proteins with N and O-linked glycans present throughout the parasite’s secretory pathway, suggesting potential shared antigens with cancer cells [120]. Moreover, the enzyme required for mucin-type O-glycosylation has been characterized in *T. gondii*, further supporting the potential shared antigens between *T. gondii* and cancer cells [121]. Therefore, the abundance of glycosylated antigens in *T. gondii* reinforces the argument for it being a cancer suppressor rather than a cancer inducer [120].

Furthermore, detailed transcriptome-sequencing data revealed that *T. gondii* infection can impact gene expression related to colorectal cancer, non-small cell lung cancer, breast cancer, and hematological cancer pathways, potentially leading to the suppression of tumor growth [38, 122]. The dense granular protein 16 (TgGRA16) has been shown to inhibit telomerase activity, leading to apoptosis in human colorectal cancer cells (HCT116) by reducing the expression of human telomerase reverse transcriptase, resulting in telomere shortening due to telomerase inactivation. This process is facilitated by the activation of tension homolog and the tumor suppressor phosphatase [123].

Interestingly, *T. gondii* and its lysate antigen have been found to help overcome drug resistance in multidrug-resistant gastric cancer and murine lymphoma cancer cell lines, such as Mdr L 5718 and Par 5718, by increasing drug accumulation through interference with the drug efflux pump that relies on ATP [124].

Similarly, the potential applications of other pathogens, such as viruses and bacteria, in the treatment of brain tumors have also been explored. The Glioma International Case-Control Study found a lower risk of glioma in individuals with a history of chickenpox from data across five countries [125]. In addition, virotherapy shows promise for glioblastoma treatment by combining cytotoxic and immunomodulatory effects [126]. Various oncolytic viruses, including herpes simplex virus type-1 [127–129], Reovirus [130], Adenovirus [131], Myxoma virus [132, 133], Poliovirus [134] and Newcastle Disease Virus [135], have been tested in clinical trials for glioma treatment. Bacteria are also being explored in brain tumor therapy, with engineered bacteria and their derivatives, like bacterial proteins and spores, showing promise for targeted drug delivery [136–138]. However, challenges and safety concerns must be addressed before bacteriotherapy can be clinically translated for applicable brain tumor therapies [139].

Interestingly, several preclinical studies have demonstrated that *T. gondii* has anti-cancer properties beyond brain tumors. Both live *Toxoplasma* and genetically attenuated strains as well as derived *T. gondii* molecules, exhibit anti-cancer activity on a range of cancer types in pre-clinical research [40]. For instance, in vitro studies have demonstrated that live *T. gondii* tachyzoites RH strain, lysate antigens, and derived *T. gondii* molecules, such as GRA15, rGRA8, GRA16, and profilin-like protein, suppressed proliferation and induced apoptosis of numerous cancer cell lines, such as colorectal cancer (HCT116) [123, 140], breast cancer (TUBO & MCF-7) [141, 142], prostate cancer (DU-145) [143], esophageal squamous cell carcinoma (EC109) [143], gastric cancer (BGC-823) [124, 144], non-small-cell lung cancer (A549) [143], chronic myeloid leukemia (K562) [145], lymphoma (Mdr L 5718 and Par 5718) [124], mastocytoma (P815) [146], fibrosarcoma (WEHI-164) [147], hepatocellular carcinoma (HepG2, Hepa1-6 & H7402) [106, 148, 149], and ovarian cancer (A2780-CP) [150].

The antineoplastic activity of *T. gondii* was further documented in vivo testing against various cancer models. For instance, infection of mice with cysts of *T. gondii* ME49 strain inhibited melanoma [97], Lewis lung carcinoma (LLC) [114], breast cancer, leukemia, and sarcoma [151] in the murine models. Similar anti-neoplastic activity was also documented following mice infection with attenuated *Toxoplasma*, such as ΔGRA17 attenuated *Toxoplasma* strain, avirulent uracil auxotroph vaccine strain, attenuated *Toxoplasma* NRTUA strain, gamma-irradiated *T. gondii* ME49 strain, ultraviolet irradiated *Toxoplasma* tachyzoites, or lactate dehydrogenase-lacking *Toxoplasma* against melanoma [152–154], LLC [152], colon adenocarcinoma [152], pancreatic cancer [155–158], and breast cancer [159] in murine models.

A safer and similar antineoplastic activity was obtained following an alternative approach using *Toxoplasma* antigens instead of live or attenuated parasites, which carries the risk of inducing infection, particularly in immunosuppressed individuals like cancer patients. Interestingly, frequent frozen and thawed tachyzoites (RH strain), lysate antigen, excretory–secretory antigen, *T. gondii* fraction A1 and C14, and formalin-fixed tachyzoite suppressed fibrosarcoma [160, 161], melanoma [162, 163], colon cancer [164], lung [165, 166], and lymphoma [146, 167, 168] in the murine models.

This raised interest in exploring which *T. gondii* molecules have the power to have potent anti-neoplastic activity. Research studies have demonstrated that recombinant profilin-like protein, GRA16, GRA15, GRA8, rGRA6Nt, as well as exosomes derived from *Toxoplasma* ME-49 infected dendritic cells, decreased tumor growth in the colon [105, 140, 169, 170], lung [166], hepatocellular carcinoma [106, 148], fibrosarcoma [171, 172], and pancreatic cancers [173] in murine models.

Interestingly, in 2024, a research study explored the relationship between *T. gondii* infection and tumorigenesis. The study found that tumor-induced immune suppression led to increased *T. gondii* replication in the brain, resulting in larger and more numerous brain cysts. This suggests that the presence of a tumor promotes the growth of *T. gondii* in the brain. Additionally, the study showed that acute and chronic *T. gondii* infections had different effects on tumor development in a murine model of LLC. Acute *T. gondii* infection, whether before or after tumor cell inoculation, decreased tumor growth by reducing bioluminescent signal, tumor weight, and size. This was accompanied by an increase in CD4^+^ and CD8^+^ T cell numbers in the TME, including cytotoxic CD8^+^ T cells and Th1 cells. In contrast, chronic *T. gondii* infection enhanced tumor growth by increasing the rate of tumor formation in the murine model [174].

It is worth mentioning that other parasites, such as *Schistosoma mansoni*, *Echinococcus granulosus*, *Trichinella spiralis*, *Trypanosoma cruzi*, *Plasmodium spp*, *Neospora caninum*, among others, have also shown similar anti-neoplastic properties by modulating host immunity and suppressing tumor development in pre-clinical studies [40]. These findings highlight the potential therapeutic value of parasites and their derived molecules as potential allies to fight cancer.

Based on the aforementioned data, human epidemiological studies have not definitively proven a causal link between *T. gondii* and the development of brain cancer or other types of cancer. On the contrary, a plethora of pre-clinical studies suggest that *T. gondii* is a promising potent anti-neoplastic tool.

### Future directions, challenges and translational limitations of the anti-neoplastic capabilities of *T.gondii*

*Toxoplasma gondii* could be a wealthy fabric for experimental trials in oncology due to its ability to reactivate the immune response, alter the TME to become immunoreactive, induce apoptosis, exhibit anti-angiogenic properties, and overcome drug resistance, a significant challenge in cancer therapy. In addition, recent research has even highlighted the anti-neoplastic properties of anti-*T.gondii* antibodies, paving the way for the development of therapeutic antibodies for targeted cancer therapy [175]. These findings offer a new perspective on how infectious agents such as *T. gondii* could potentially be utilized in the future to fight brain tumors.

However, clinical translation of *T.gondii-*based cancer therapy faces obstacles such as ethical concerns, lack of safety data on attenuated *T.gondii* strains and the risk of reversion to virulence. Challenges also include difficulties in translating results from animal models to humans, the parasite’s need for living host cells complicates large-scale cultivation, and the excessive inflammatory responses that may promote tumor progression [176]. New approaches involve using *T. gondii-derived* components like GRA16, GRA8, profilin-like protein, rGRA6Nt protein, and exosomes from *T. gondii*-infected dendritic cells and modern technology could enhance therapy efficacy, enable standardized production, and provide a solid foundation for advancing promising pre-clinical studies into human trials. This could offer renewed hope for cancer therapy.

## Conclusion

Epidemiological studies have not yet provided firm evidence of a causal relationship between *T. gondii* infection and brain cancer development. In data analysis, the presence of toxoplasmosis as an opportunistic infection in cancer patients should be carefully considered. Interestingly, experimental studies have shown the unique role of *T. gondii* in modifying the tumor microenvironment from being immunosuppressive to immunoreactive. *Toxoplasma gondii* has various mechanisms to combat cancer, such as immunomodulation, induction of apoptosis, anti-angiogenesis, and molecular mimicry with cancer cells. This review highlights the importance of exploring the potential of *T. gondii* as a valuable resource for its promising anti-neoplastic activity on brain cancer and other types of cancer.

## Data Availability

No datasets were generated or analysed during the current study.

## References

[1] Sung H, et al. Global cancer statistics 2020: GLOBOCAN estimates of incidence and mortality worldwide for 36 cancers in 185 countries. CA Cancer J Clin. 2021;71(3):209–49. 33538338 10.3322/caac.21660

[2] Gersten O, Wilmoth JR. The cancer transition in Japan since 1951. Demogr Res. 2002;7:271–306.

[3] Omram AR. The epidemiologic transition: a theory of the epidemiology of population change. Milbank Mem Fund. 2001;79(2):161–70. 5155251

[4] Islami F, Torre LA, Jemal A. Global trends of lung cancer mortality and smoking prevalence. Transl Lung Cancer Res. 2015;4(4):327. 26380174 10.3978/j.issn.2218-6751.2015.08.04 PMC4549470

[5] Ostrom QT, et al. CBTRUS statistical report: primary brain and other central nervous system tumors diagnosed in the United States in 2015–2019. Neurooncology. 2022;24(Supplement5):v1–95. 10.1093/neuonc/noac202 PMC9533228 36196752

[6] Price T, Goetz K, Lovell M. Neuropsychiatric aspects of brain tumors. Am J Psychiatry. 2007: The American Psychiatric Publishing Textbook of Neuropsychiatry and Behavioral Neurosciences, Fifth Edition. 735 – 64. 10.1176/appi.ajp.2007.07101662 22706504

[7] Madhusoodanan S, et al. Psychiatric aspects of brain tumors: A review. World J Psychiatry. 2015;5(3):273. 26425442 10.5498/wjp.v5.i3.273 PMC4582304

[8] Litofsky NS, Resnick AG. The relationships between depression and brain tumors. J Neurooncol. 2009;94(2):153–61. 19262993 10.1007/s11060-009-9825-4

[9] Spence SA, Taylor DG, Hirsch SR. Depressive disorder due to craniopharyngioma. J R Soc Med. 1995;88(11):637–8. 8544149 10.1177/014107689508801109 PMC1295388

[10] Wellisch DK, et al. Predicting major depression in brain tumor patients. Psychooncology. 2002;11(3):230–8. 12112483 10.1002/pon.562

[11] Kumar GS, et al. Role of imaging in CNS infections. Indian J Pathol Microbiol. 2022;65(Suppl 1):S153–63. 35562146 10.4103/ijpm.ijpm_1162_21

[12] Tandel GS, et al. Role of ensemble deep learning for brain tumor classification in multiple magnetic resonance imaging sequence data. Diagnostics. 2023;13(3):481. 36766587 10.3390/diagnostics13030481 PMC9914433

[13] Barisano G, et al. Complications of radiotherapy and radiosurgery in the brain and spine. Neurographics (2011). 2018;8(3):167–187. 10.3174/ng.1700066 PMC8981962 35388375

[14] de Martel C, et al. Global burden of cancer attributable to infections in 2018: a worldwide incidence analysis. Lancet Glob Health. 2020;8(2):e180–90. 31862245 10.1016/S2214-109X(19)30488-7

[15] Kato I, Zhang J, Sun J. Bacterial-viral interactions in human orodigestive and female genital tract cancers: A summary of epidemiologic and laboratory evidence. Cancers. 2022;14(2):425. 35053587 10.3390/cancers14020425 PMC8773491

[16] van Elsland D, Neefjes J. Bacterial infections and cancer. EMBO Rep. 2018;19(11):e46632. 30348892 10.15252/embr.201846632 PMC6216254

[17] Magon KL, Parish JL. From infection to cancer: how DNA tumour viruses alter host cell central carbon and lipid metabolism. Open Biol. 2021;11(3):210004. 33653084 10.1098/rsob.210004 PMC8061758

[18] Fasihi-Karami M, et al. Association between some helminths and tumorigenesis through immunological and biochemical factors. Curr Cancer Ther Rev. 2023;19(2):96–102.

[19] Elrefaey S, et al. HPV in oropharyngeal cancer: the basics to know in clinical practice. Acta Otorhinolaryngol Ital. 2014;34(5):299. 25709145 PMC4299160

[20] Ramagosa R, et al. Human papillomavirus infection and ultraviolet light exposure as epidermoid inclusion cyst risk factors in a patient with epidermodysplasia verruciformis? J Am Acad Dermatol. 2008;58(5):S68. e6. 10.1016/j.jaad.2007.01.032 PMC2587233 18489051

[21] Kew MC. Synergistic interaction between aflatoxin B1 and hepatitis B virus in hepatocarcinogenesis. Liver Int. 2003;23(6):405–9. 14986813 10.1111/j.1478-3231.2003.00869.x

[22] Rahman MM, et al. Microbiome in cancer: Role in carcinogenesis and impact in therapeutic strategies. Biomed Pharmacother. 2022;149:112898. 35381448 10.1016/j.biopha.2022.112898

[23] Zhao L-Y, et al. Role of the gut microbiota in anticancer therapy: from molecular mechanisms to clinical applications. Sig Transduct Target Ther. 2023;8(1):201. 10.1038/s41392-023-01406-7 PMC10183032 37179402

[24] Wyss K, et al. Malaria and risk of lymphoid neoplasms and other cancer: a nationwide population-based cohort study. BMC Med. 2020;18(1):296. 33121475 10.1186/s12916-020-01759-8 PMC7596993

[25] Montoya J, Liesenfeld O. Toxoplasmosis. Lancet. 2004;363:1965–1976. . 10.1016/S0140-6736(04)16412-X 15194258

[26] Tenter AM, Heckeroth AR, Weiss LM. *Toxoplasma gondii*: from animals to humans. Int J Parasitol. 2000;30(12–13):1217–58. 11113252 10.1016/s0020-7519(00)00124-7 PMC3109627

[27] Dubey J, Lindsay D, Speer C. Structures of *Toxoplasma gondii* tachyzoites, bradyzoites, and sporozoites and biology and development of tissue cysts. Clin Microbiol Rev. 1998;11(2):267–99. 9564564 10.1128/cmr.11.2.267 PMC106833

[28] Jung B-K, et al. Exosomal miRNA-21 from *Toxoplasma gondii*-infected microglial cells induces the growth of U87 glioma cells by inhibiting tumor suppressor genes. Sci Rep. 2022;12(1):16450. 36180486 10.1038/s41598-022-20281-w PMC9525672

[29] El Skhawy N, Eissa MM. Shedding light on a mysterious link between *Toxoplasma gondii* and cancer: a review. Exp Parasitol. 2023;250:108544. 37149210 10.1016/j.exppara.2023.108544

[30] Ryan P, et al. Tumours of the brain and presence of antibodies to *Toxoplasma gondii*. Int J Epidemiol. 1993;22(3):412–9. 8359956 10.1093/ije/22.3.412

[31] Thomas F, et al. Incidence of adult brain cancers is higher in countries where the protozoan parasite *Toxoplasma gondii* is common. Biol Lett. 2012;8(1):101–3. 21795265 10.1098/rsbl.2011.0588 PMC3259962

[32] Vittecoq M, et al. Brain cancer mortality rates increase with *Toxoplasma gondii* seroprevalence in France. Infect Genet Evol. 2012;12(2):496–8. 22285308 10.1016/j.meegid.2012.01.013

[33] Abdollahi A, et al. *Toxoplasma gondii* infection/exposure and the risk of brain tumors: A systematic review and meta-analysis. Cancer Epidemiol. 2022;77:102119. 35152168 10.1016/j.canep.2022.102119

[34] Asgari Q, et al. *Toxoplasma gondii* infection in patients with brain tumors in Southern Iran: a case-control study. J Parasit Dis. 2023;47(2):291–6. 37193506 10.1007/s12639-022-01541-y PMC10182190

[35] Hamouda MM, et al. *Toxoplasma gondii*: Seroprevalence and association with childhood brain tumors in Egypt. Acta Trop. 2024;251:107123. 38242223 10.1016/j.actatropica.2024.107123

[36] Jung B-K, et al. High *Toxoplasma gondii* seropositivity among brain tumor patients in Korea. Kor J Parasitol. 2016;54(2):201. 10.3347/kjp.2016.54.2.201 PMC4870975 27180580

[37] Nguyen YT, et al. *Toxoplasma gondii* infection supports the infiltration of T cells into brain tumors. J Neuroimmunol. 2024;393:578402. 38996717 10.1016/j.jneuroim.2024.578402 PMC11318612

[38] Seyedeh MS, et al. Low titer of antibody against *Toxoplasma gondii* may be related to resistant to cancer. J Cancer Res Ther. 2015;11(2):305–7. 26148590 10.4103/0973-1482.144638

[39] El Khadrawe MT, Skhawy NE, Eissa MM. Relationship between parasites and lung cancer: Unveiling the link. Trop Med Int Health. 2025;30(7):613–24. 40341730 10.1111/tmi.14119

[40] Eissa MM, Salem AE, El Skhawy N. Parasites revive hope for cancer therapy. Eur J Med Res. 2024;29(1):489. 39367471 10.1186/s40001-024-02057-2 PMC11453045

[41] Schuman LM, Choi N, Gullen W. Relationship of central nervous system neoplasms to *Toxoplasma gondii* infection. Am J Public Health Nations Health. 1967;57(5):848–56. 6067209 10.2105/ajph.57.5.848 PMC1227362

[42] Cong W, et al. *Toxoplasma gondii* infection in cancer patients: prevalence, risk factors, genotypes and association with clinical diagnosis. Cancer Lett. 2015;359(2):307–13. 25641340 10.1016/j.canlet.2015.01.036

[43] Mostafa NES, et al. The relationship between toxoplasmosis and different types of human tumors. J Infect Dev Ctries. 2018;12(02):137–41. 31825916 10.3855/jidc.9672

[44] Hodge JM, et al. *Toxoplasma gondii* infection and the risk of adult glioma in two prospective studies. Int J Cancer. 2021;148(10):2449–56. 33427315 10.1002/ijc.33443

[45] Benson V, Green J, Beral V. The relationship between owning a cat and the risk of developing a brain cancer in a prospective study of UK women: comment on Thomas. Biol Lett. 2012;8(6):1040–1. 22915632 10.1098/rsbl.2012.0511 PMC3497111

[46] Embil JA, et al. Visualization of *Toxoplasma gondii* in the cerebrospinal fluid of a child with a malignant astrocytoma. Can Med Assoc J. 1985;133(3):213–4. 4016628 PMC1346154

[47] Aabasian L, et al. Hormonal changes in women with breast cancer infected with *Toxoplasma gondii*. J Bas Res Med Sci. 2016;3(1):16–21.

[48] Alim M, Ozcelik S, Ozpinar N. Seroprevalence of *Toxoplasma gondii* in patients receiving cancer treatment. Cumhur Med. 2018;40(1):19–24.

[49] Fadel EF, et al. Serological and molecular detection of *Toxoplasma gondii* among cancer patients in Sohag, Upper Egypt: a case-control study. Sci Rep. 2025;15(1):5236. 39939648 10.1038/s41598-025-88680-3 PMC11822034

[50] Khabaz MN, Elkhateeb L, Al-Alami J. Reactivation of latent *Toxoplasma gondii* in immunocompromised cancer patients. Comp Clin Pathol. 2011;20(2):183–6.

[51] Khalil H, et al. Opportunistic parasitic infections in immunocompromised hosts. J Egypt Soc Parasitol. 1991;21(3):657–68. 1765676

[52] Jančálek R, et al. Oportunní infekce mozku u pacientů po komplexní terapii nádorového onemocnění. klinická onkologie, 2011:46. 21542275

[53] Malek RA, et al. Toxoplasmosis an overlooked disease: seroprevalence in cancer patients. Asian Pac J Cancer Prev. 2018;19(7):1987. 10.22034/APJCP.2018.19.7.1987 PMC6165665 30051689

[54] Wang B, et al. Investigation of anti-*Toxoplasma* gondii antibodies in immunodeficient patients. Zhongguo ji Sheng Chong xue yu ji Sheng Chong Bing za zhi=. Chin J Parasitol Parasitic Dis. 2000;18(4):224–6. 12567666

[55] Wang L, et al. Seroprevalence and genetic characterization of *Toxoplasma gondii* in cancer patients in Anhui Province. East China Parasit Vectors. 2015;8(1):162. 10.1186/s13071-015-0778-5 PMC4379604 25889184

[56] Wang Z-D, et al. *Toxoplasma gondii* infection in immunocompromised patients: a systematic review and meta-analysis. Front Microbiol. 2017;8:389. 28337191 10.3389/fmicb.2017.00389 PMC5343064

[57] Wiercińska-Drapało A, et al. *Toksoplazmoza* mózgu jako przykład inwazji oporutunistycznej u kobiety żyjącej z HIV. Wiadomości Parazytologiczne. 1999;45(3):401–3. 16886384

[58] Conley FK, Remington JS. Nonspecific inhibition of tumor growth in the central nervous system: observations of intracerebral ependymoblastoma in mice with chronic *Toxoplasma* infection. J Natl Cancer Inst. 1977;59(3):963–73. 894752 10.1093/jnci/59.3.963

[59] Choo J-D, et al. Inhibitory effects of *Toxoplasma* antigen on proliferation and invasion of human glioma cells. J Korean Neurosurg Soc. 2005;37(2):129–36.

[60] Nguyen Y, et al. Harness the immune-modulatory activities of *Toxoplasma gondii* to improve lymphocyte infiltration into brain tumors. Cancer Immunol Res. 2022;10(1Supplement):P040–040.

[61] Abdoli A, et al. Screening of toxoplasmosis in cancer patients: a concern. Trop Doct. 2019;49(1):31–4. 30270766 10.1177/0049475518801618

[62] Bajnok J, et al. High frequency of infection of lung cancer patients with the parasite *Toxoplasma gondii*. ERJ open research. 2019;5(2). 10.1183/23120541.00143-2018 PMC6536861 31149623

[63] Yu Y, et al. Increased risk of *Toxoplasma gondii* infection in patients with colorectal cancer in eastern china: seroprevalence, risk factors, and a case–control study. BioMed Res Int. 2020;2020(1):2539482. 33083457 10.1155/2020/2539482 PMC7563061

[64] Ahmed DF, Saheb EJ. The association of *Toxoplasma gondii* infection in breast and colorectal cancer patients. Int J Clin Oncol. 2017;2:86–92.

[65] Kalantari N, et al. Detection of *Toxoplasma gondii* DNA in malignant breast tissues in breast cancer patients. IJMCM. 2017;6(3):190. 29682491 10.22088/acadpub.BUMS.6.3.190 PMC5898643

[66] Mahmood AA, Rezaig ZS. Detection of *Toxoplasma gondii* in Iraqi women with breast cancer by real-time PCR. Kirkuk Univ J Sci Stud. 2019;14(3):1–9.

[67] Zhou N, et al. Seroprevalence and risk factors of *Toxoplasma gondii* infection in oral cancer patients in China: a case–control prospective study. Epidemiol Infect. 2018;146(15):1891–5. 30001756 10.1017/S0950268818001978 PMC6452986

[68] Hosseini SA, et al. Toxoplasmosis among cancer patients undergoing chemotherapy: a population study based on the serological, molecular and epidemiological aspects. Trans R Soc Trop Med Hyg. 2021;115(6):677–86. 33130887 10.1093/trstmh/traa112

[69] Ali MI, et al. *Toxoplasma gondii* in cancer patients receiving chemotherapy: seroprevalence and interferon gamma level. J Parasit Dis. 2019;43(3):464–71. 31406412 10.1007/s12639-019-01111-9 PMC6667530

[70] Imam A, et al. Serologic evidence of *Toxoplasma gondii* infection among cancer patients. A prospective study from Qassim region, Saudi Arabia. Saudi Med J. 2017;38(3):319. 28251231 10.15537/smj.2017.3.18535 PMC5387912

[71] Hijjawi NS et al. Seroprevalence of *Toxoplasma gondii* in cancer patients admitted to hospitals of the royal medical services in Jordan. Jordan J Biol Sci. 2018;11(5).

[72] Al-Tameemi IA, Abdullah BH, Raisan SJ. Seroprevalence of *Toxoplasma gondii* among cancer patients in Basrah province/Iraq. World J Pharm Res. 2018;8(1):193–9.

[73] Jiang C, et al. The seroprevalence of *Toxoplasma gondii* in Chinese population with cancer: a systematic review and meta-analysis. Medicine. 2015;94(50):e2274. 26683951 10.1097/MD.0000000000002274 PMC5058923

[74] Sauer TC, et al. Primary intraocular (retinal) lymphoma after ocular toxoplasmosis. Retin Cases Brief Rep. 2010;4(2):160–3. 20454548 10.1097/ICB.0b013e3181ad3916 PMC2865161

[75] Knoll LJ, et al. Pearls collections: What we can learn about infectious disease and cancer. Public Library of Science San Francisco, CA USA; 2018. p. e1006915. 10.1371/journal.ppat.1006915 PMC5875890 29596508

[76] Caner A. Toxoplasma gondii could have a possible role in the cancer mechanism by modulating the host’s cell response. Acta Trop. 2021;220:105966. 34023305 10.1016/j.actatropica.2021.105966

[77] Ngô HM, et al. *Toxoplasma* modulates signature pathways of human epilepsy, neurodegeneration & cancer. Sci Rep. 2017;7(1):11496. 28904337 10.1038/s41598-017-10675-6 PMC5597608

[78] Blader IJ, Manger ID, Boothroyd JC. Microarray analysis reveals previously unknown changes in *Toxoplasma gondii*-infected human cells. J Biol Chem. 2001;276(26):24223–31. 11294868 10.1074/jbc.M100951200

[79] Gail M, Gross U, Bohne W. Transcriptional profile of *Toxoplasma gondii* infected human fibroblasts as revealed by gene-array hybridization. Mol Genet Genomics. 2001;265(5):905–12. 11523808 10.1007/s004380100487

[80] Okomo-Adhiambo M, Beattie C, Rink A. cDNA microarray analysis of host-pathogen interactions in a porcine in vitro model for *Toxoplasma gondii* infection. Infect Immun. 2006;74(7):4254–65. 16790800 10.1128/IAI.00386-05 PMC1489723

[81] Pang JC-s, et al. Oncogenic role of microRNAs in brain tumors. Acta Neuropathol. 2009;117(6):599–611. 19343354 10.1007/s00401-009-0525-0

[82] Turner JD, et al. The many roles of microRNAs in brain tumor biology. Neurosurg Focus. 2010;28(1):E3. 20043718 10.3171/2009.10.FOCUS09207

[83] Wang D, et al. Human microRNA oncogenes and tumor suppressors show significantly different biological patterns: from functions to targets. PLoS ONE. 2010;5(9):e13067. 20927335 10.1371/journal.pone.0013067 PMC2948010

[84] Krichevsky AM, et al. A microRNA array reveals extensive regulation of microRNAs during brain development. RNA. 2003;9(10):1274–81. 13130141 10.1261/rna.5980303 PMC1370491

[85] Soreq H, Wolf Y. NeurimmiRs: microRNAs in the neuroimmune interface. Trends Mol Med. 2011;17(10):548–55. 21813326 10.1016/j.molmed.2011.06.009

[86] Kranenburg O. The KRAS oncogene: past, present, and future. Biochim Biophys Acta. 2005;1756(2):81–2. 16269215 10.1016/j.bbcan.2005.10.001

[87] Zou H, et al. Apaf-1, a human protein homologous to C. elegans CED-4, participates in cytochrome c–dependent activation of caspase-3. Cell. 1997;90(3):405–13. 9267021 10.1016/s0092-8674(00)80501-2

[88] Kalantari N, et al. Association between *Toxoplasma gondii* exposure and paediatrics haematological malignancies: a case–control study. Epidemiol Infect. 2018;146(15):1896–902. 30092850 10.1017/S0950268818002194 PMC6452987

[89] Zhou N, et al. Seroprevalence and risk factors of *Toxoplasma gondii* infection in children with leukemia in Shandong Province, Eastern China: a case—control prospective study. PeerJ. 2019;7:e6604. 30886781 10.7717/peerj.6604 PMC6420808

[90] Arefkhah N, et al. Seroprevalence and risk factors of *Toxoplasma gondii* infection among cancer and hemodialysis patients in southwest Iran. Clin Epidemiol Glob Health. 2019;7(4):596–9.

[91] Bo R, et al. Co-existence of hepatocellular carcinoma and cystic echinococcosis. Infect Agents Cancer. 2020;15(1):5. 10.1186/s13027-020-0275-0 PMC6988235 32010203

[92] Plumelle Y, et al. Effect of *Strongyloides stercoralis* infection and eosinophilia on age at onset and prognosis of adult T-cell leukemia. Am J Clin Pathol. 1997;107(1):81–7. 8980372 10.1093/ajcp/107.1.81

[93] Liu J, et al. Observation of *Trichinella* on C6 glioma in BALB/c mice. J Apoplexy Nerv Dis. 2008;6:722–4.

[94] Parker WB, et al. The use of Trichomonas vaginalis purine nucleoside phosphorylase to activate fludarabine in the treatment of solid tumors. Cancer Chemother Pharmacol. 2020;85(3):573–83. 31915968 10.1007/s00280-019-04018-7 PMC7039746

[95] Salanti A, et al. Targeting human cancer by a glycosaminoglycan binding malaria protein. Cancer Cell. 2015;28(4):500–14. 26461094 10.1016/j.ccell.2015.09.003 PMC4790448

[96] Tao Z, et al. Preclinical study of Plasmodium immunotherapy combined with radiotherapy for solid tumors. Cells. 2022;11(22):3600. 36429033 10.3390/cells11223600 PMC9688403

[97] Hunter CA, et al. Cutting edge: systemic inhibition of angiogenesis underlies resistance to tumors during acute toxoplasmosis. J Immunol. 2001;166(10):5878–81. 11342601 10.4049/jimmunol.166.10.5878

[98] Park E, et al. *Toxoplasma gondii* infection drives conversion of NK cells into ILC1-like cells. elife. 2019;8:e47605. 31393266 10.7554/eLife.47605 PMC6703900

[99] Chen J, Liao W, Peng H. *Toxoplasma gondii* infection possibly reverses host immunosuppression to restrain tumor growth. Front Cell Infect Microbiol. 2022;12:959300. 36118042 10.3389/fcimb.2022.959300 PMC9470863

[100] Eissa MM, et al. Prophylactic antineoplastic activity of *Toxoplasma gondii* RH derived antigen against Ehrlich solid carcinoma with evidence of shared antigens by comparative immunoblotting. Infect Agents Cancer. 2023;18(1):21. 10.1186/s13027-023-00500-3 PMC10082516 37029378

[101] Ismail CA, et al. *Toxoplasma gondii*-derived antigen modifies tumor microenvironment of Ehrlich solid carcinoma murine model and enhances immunotherapeutic activity of cyclophosphamide. Med Oncol. 2023;40(5):136. 37014499 10.1007/s12032-023-01994-y PMC10073061

[102] Eissa MM, et al. Unveiling the anti-neoplastic potential of *Schistosoma mansoni*-derived antigen against breast cancer: a pre-clinical study. Eur J Med Res. 2025;30(1):304. 40247360 10.1186/s40001-025-02531-5 PMC12007238

[103] Eissa MM, et al. Immuno-therapeutic potential of *Schistosoma mansoni* and *Trichinella spiralis* antigens in a murine model of colon cancer. Invest New Drugs. 2019;37(1):47–56. 29808307 10.1007/s10637-018-0609-6

[104] Wang X, et al. *Toxoplasma* rhoptry proteins that affect encephalitis outcome. Cell Death Discov. 2023;9(1):439. 38049394 10.1038/s41420-023-01742-1 PMC10696021

[105] Mani R, et al. A novel protozoa parasite-derived protein adjuvant is effective in immunization with cancer cells to activate the cancer-specific protective immunity and inhibit the cancer growth in a murine model of colorectal cancer. Cells. 2024;13(2):111. 38247803 10.3390/cells13020111 PMC10814441

[106] Li Y, et al. Macrophages polarized by expression of ToxoGRA15II inhibit growth of hepatic carcinoma. Front Immunol. 2017;8:137. 28243242 10.3389/fimmu.2017.00137 PMC5303709

[107] Pyo K-H, et al. Immune adjuvant effect of a *Toxoplasma gondii* profilin-like protein in autologous whole-tumor-cell vaccination in mice. Oncotarget. 2016;7(45):74107. 27687589 10.18632/oncotarget.12316 PMC5342039

[108] Eissa MM, El-Faham MH, El Skhawy N. Bridging the gap for diverse applications of parasites as advanced cancer therapeutics: current progress and future directions. Infect Agents Cancer. 2025;20:53. 10.1186/s13027-025-00679-7 PMC12309174 40731364

[109] Ahmadpour E, et al. Overview of apoptosis, autophagy, and inflammatory processes in *Toxoplasma gondii* infected cells. Pathogens. 2023;12(2):253. 36839525 10.3390/pathogens12020253 PMC9966443

[110] Payne TM, Molestina RE, Sinai AP. Inhibition of caspase activation and a requirement for NF-κB function in the *Toxoplasma gondii-*mediated blockade of host apoptosis. J Cell Sci. 2003;116(21):4345–58. 12966169 10.1242/jcs.00756

[111] Al-Bedeary S, Getta HA, Al-Sharafi D. The hallmarks of cancer and their therapeutic targeting in current use and clinical trials. Iraqi J Hematol. 2020;9(1):1–10.

[112] Lopes-Coelho F, et al. Anti-angiogenic therapy: current challenges and future perspectives. Int J Mol Sci. 2021;22(7):3765. 33916438 10.3390/ijms22073765 PMC8038573

[113] Hafez EN, et al. Gamma radiation-attenuated *Toxoplasma gondii* provokes apoptosis in Ehrlich ascites carcinoma-bearing mice generating long-lasting immunity. Technol Cancer Res. 2020;19:1533033820926593. 10.1177/1533033820926593 PMC7309383 32567499

[114] Kim J-O, et al. Inhibition of Lewis lung carcinoma growth by *Toxoplasma gondii* through induction of Th1 immune responses and inhibition of angiogenesis. J Kor Med Sci. 2007;22(Suppl):S38. 10.3346/jkms.2007.22.S.S38 PMC2694397 17923753

[115] Darani HY, Yousefi M. Parasites and cancers: parasite antigens as possible targets for cancer immunotherapy. Future Oncol. 2012;8(12):1529–35. 23231515 10.2217/fon.12.155

[116] Eissa MM, et al. Molecular mimicry between parasites and cancer: a novel approach for developing cancer vaccines and therapeutic antibodies. Cancer Immunol Immunother. 2025;74(7):212. 40402283 10.1007/s00262-025-04069-1 PMC12098237

[117] Mohamadi F, et al. Anti-*Toxoplasma gondii* antibodies attach to mouse cancer cell lines but not normal mouse lymphocytes. Biomed Rep. 2019;1(01):1–6. 10.3892/br.2019.1186 PMC6403471 30906547

[118] Hosseini F, et al. Human anti-*Toxoplasma* antibodies attach strongly to breast cancer cells. Int J Cancer Manag. 2023;16(1):183–8.

[119] Osinaga E. Expression of cancer-associated simple mucin‐type O‐glycosylated antigens in parasites. IUBMB Life. 2007;59(4–5):269–73. 17505964 10.1080/15216540601188553

[120] Luk FC, Johnson TM, Beckers CJ. N-linked glycosylation of proteins in the protozoan parasite *Toxoplasma gondii*. Mol Biochem Parasitol. 2008;157(2):169–78. 18096254 10.1016/j.molbiopara.2007.10.012 PMC2258246

[121] Wojczyk BS, et al. cDNA cloning and expression of UDP-N-acetyl-D-galactosamine: polypeptide N-acetylgalactosaminyltransferase T1 from *Toxoplasma gondii*. Mol Biochem Parasitol. 2003;131(2):93–107. 14511808 10.1016/s0166-6851(03)00196-8

[122] Lu G, et al. Transcriptome sequencing investigated the tumor-related factors changes after *T. gondii* infection. Front Microbiol. 2019;10:181. 30792708 10.3389/fmicb.2019.00181 PMC6374557

[123] Seo S-H, et al. PTEN/AKT signaling pathway related to hTERT downregulation and telomere shortening induced in Toxoplasma GRA16-expressing colorectal cancer cells. Biomed Pharmacother. 2022;153:113366. 35810694 10.1016/j.biopha.2022.113366

[124] Varga A, et al. *Toxoplasma* infection and cell free extract of the parasites are able to reverse multidrug resistance of mouse lymphoma and human gastric cancer cells in vitro. Anticancer Res. 1999;19(2A):1317–24. 10368693

[125] Amirian ES, et al. History of chickenpox in glioma risk: a report from the glioma international case–control study (GICC). Cancer Med. 2016;5(6):1352–8. 26972449 10.1002/cam4.682 PMC4924393

[126] Hamad A, et al. Recent developments in glioblastoma therapy: oncolytic viruses and emerging future strategies. Viruses. 2023;15(2):547. 36851761 10.3390/v15020547 PMC9958853

[127] Markert J, et al. Conditionally replicating herpes simplex virus mutant, G207 for the treatment of malignant glioma: results of a phase I trial. Gene Ther. 2000;7(10):867–74. 10845725 10.1038/sj.gt.3301205

[128] Markert JM, et al. Phase Ib trial of mutant herpes simplex virus G207 inoculated pre-and post-tumor resection for recurrent GBM. Mol Ther. 2009;17(1):199–207. 18957964 10.1038/mt.2008.228 PMC2834981

[129] Markert JM, et al. A phase 1 trial of oncolytic HSV-1, G207, given in combination with radiation for recurrent GBM demonstrates safety and radiographic responses. Mol Ther. 2014;22(5):1048–55. 24572293 10.1038/mt.2014.22 PMC4015243

[130] Kicielinski KP, et al. Phase 1 clinical trial of intratumoral reovirus infusion for the treatment of recurrent malignant gliomas in adults. Mol Ther. 2014;22(5):1056–62. 24553100 10.1038/mt.2014.21 PMC4015229

[131] Chiocca EA, et al. A phase I open-label, dose-escalation, multi-institutional trial of injection with an E1B-Attenuated adenovirus, ONYX-015, into the peritumoral region of recurrent malignant gliomas, in the adjuvant setting. Mol Ther. 2004;10(5):958–66. 15509513 10.1016/j.ymthe.2004.07.021

[132] Allen C, et al. Oncolytic measles virus strains have significant antitumor activity against glioma stem cells. Gene Ther. 2013;20(4):444–9. 22914495 10.1038/gt.2012.62 PMC3509233

[133] Allen C, et al. Interleukin-13 displaying retargeted oncolytic measles virus strains have significant activity against gliomas with improved specificity. Mol Ther. 2008;16(9):1556–64. 18665158 10.1038/mt.2008.152 PMC2748750

[134] Gromeier M, et al. Intergeneric poliovirus recombinants for the treatment of malignant glioma. Proc Natl Acad Sci. 2000;97(12):6803–8. 10841575 10.1073/pnas.97.12.6803 PMC18745

[135] Freeman AI, et al. Phase I/II trial of intravenous NDV-HUJ oncolytic virus in recurrent glioblastoma multiforme. Mol Ther. 2006;13(1):221–8. 16257582 10.1016/j.ymthe.2005.08.016

[136] Chen H, et al. Biomimetic lipopolysaccharide-free bacterial outer membrane‐functionalized nanoparticles for brain‐targeted drug delivery. Adv Sci. 2022;9(16):2105854. 10.1002/advs.202105854 PMC9165477 35355446

[137] Zhou S, et al. Tumour-targeting bacteria engineered to fight cancer. Nat Rev Cancer. 2018;18(12):727–43. 30405213 10.1038/s41568-018-0070-z PMC6902869

[138] Sun R, et al. Bacteria loaded with glucose polymer and photosensitive ICG silicon-nanoparticles for glioblastoma photothermal immunotherapy. Nat Commun. 2022;13(1):5127. 36050316 10.1038/s41467-022-32837-5 PMC9433534

[139] Zhang R, Li X, Zhang S. The role of bacteria in central nervous system tumors: opportunities and challenges. Microorganisms. 2024;12(6):1053. 38930435 10.3390/microorganisms12061053 PMC11205425

[140] Kim J-S, et al. *Toxoplasma gondii* GRA8-derived peptide immunotherapy improves tumor targeting of colorectal cancer. Oncotarget. 2020;11(1):62. 32002124 10.18632/oncotarget.27417 PMC6967779

[141] Eissa MM et al. Evaluation of cytotoxic activity of live *Toxoplasma gondii* tachyzoites and toxoplasma antigen on MCF-7 human breast cancer cell line. EUREKA: Life Sci. 2022(2):45–50.

[142] Şahar EA, et al. *Toxoplasma gondii* destroys Her2/Neu-expressing mammary cancer cells in vitro using a continuous feed medium approach. J Infect Dev Ctries. 2020;14(10):1204–9. 33175718 10.3855/jidc.12820

[143] Xin W, et al. Impact of Toxoplasma gondii on the proliferation and apoptosis of tumor cell lines. Zhongguo Ji Sheng Chong Xue Yu Ji Sheng Chong Bing Za Zhi. 2012;30(2):17. 22908820

[144] Luo Q, et al. Effect of culture supernatant of *Toxoplasma gondii* on the proliferation and apoptosis of BGC-823 cells. Zhongguo ji Sheng Chong xue yu ji Sheng Chong Bing za zhi=. Chin J Parasitol Parasitic Dis. 2014;32(2):123–7. 25065211

[145] Zhang X, et al. Apoptosis of human leukemia K562 cell *in vitro* induced by *Toxoplasma gondii.* Zhongguo ji sheng chong xue yu ji sheng chong bing za zhi=. Chin J Parasitol Parasitic Dis. 2007;25(3):185–8. 18038773

[146] Miyahara K, et al. Therapeutic effects of *Toxoplasma* lysate antigen on 20-methylcholanthrene-induced BALB/c mouse tumors. J Vet Med Sci. 1992;54(1):7–12. 1558892 10.1292/jvms.54.7

[147] Shirzad H et al. *Toxoplasma gondii* but not *Leishmania major* or *Trichomonas vaginalis* decreases cell proliferation and increases cell death on fibrosarcoma cancer cells in culture medium. World J Vaccines. 2012,:2:105–108

[148] Kim SG, et al. Increase in the nuclear localization of PTEN by the *Toxoplasma* GRA16 protein and subsequent induction of p53-dependent apoptosis and anticancer effect. J Cell Mol Med. 2019;23(5):3234–45. 30834688 10.1111/jcmm.14207 PMC6484329

[149] Wang G, Gao M. Influence of *Toxoplasma gondii* on *in vitro* proliferation and apoptosis of hepatoma carcinoma H7402 cell. Asian Pac J Trop Med. 2016;9(1):63–6. 26851789 10.1016/j.apjtm.2015.12.013

[150] Elikaei A, Vazini H, Javani F. Anticancer effects of parasite extracts of leishmaniasis and *Toxoplasma* On resistant cell line (A2780-CP) and sensitive (A2780) to cisplatin. Appl Biol. 2018;31(2):5–22.

[151] Hibbs JB Jr, Lambert LH Jr, Remington JS. Resistance to murine tumors conferred by chronic infection with intracellular protozoa, *Toxoplasma gondii* and Besnoitia jellisoni. J Infect Dis. 1971;124(6):587–92. 5127071 10.1093/infdis/124.6.587

[152] Zhu Y-C, et al. Synergy between *Toxoplasma gondii* type I ∆GRA17 immunotherapy and PD-L1 checkpoint inhibition triggers the regression of targeted and distal tumors. J Immunother Cancer. 2021;9(11):e002970. 34725213 10.1136/jitc-2021-002970 PMC8562526

[153] Baird JR, et al. Immune-mediated regression of established B16F10 melanoma by intratumoral injection of attenuated *Toxoplasma gondii* protects against rechallenge. J Immunol. 2013;190(1):469–78. 23225891 10.4049/jimmunol.1201209 PMC3529845

[154] Li Y, et al. Antitumor effects of a *Toxoplasma* mutant lacking lactate dehydrogenases. Parasitol Res. 2021;120(9):3335–9. 34405281 10.1007/s00436-021-07283-9

[155] Bahwal SA, et al. Attenuated *Toxoplasma gondii* enhances the antitumor efficacy of anti-PD1 antibody by altering the tumor microenvironment in a pancreatic cancer mouse model. J Cancer Res Clin Oncol. 2022;148(10):2743–57. 35556163 10.1007/s00432-022-04036-8 PMC11800998

[156] Fox BA, et al. Targeting tumors with nonreplicating *Toxoplasma gondii* uracil auxotroph vaccines. Trends Parasitol. 2013;29(9):431–7. 23928100 10.1016/j.pt.2013.07.001 PMC3777737

[157] Sanders KL, Fox BA, Bzik DJ. Attenuated *Toxoplasma gondii* stimulates immunity to pancreatic cancer by manipulation of myeloid cell populations. Cancer Immunol Res. 2015;3(8):891–901. 25804437 10.1158/2326-6066.CIR-14-0235 PMC4526316

[158] Sanders KL, Fox BA, Bzik DJ. Attenuated *Toxoplasma gondii* therapy of disseminated pancreatic cancer generates long-lasting immunity to pancreatic cancer. Oncoimmunology. 2016;5(4):e1104447. 27141388 10.1080/2162402X.2015.1104447 PMC4839330

[159] Xu L-Q, et al. A uracil auxotroph *Toxoplasma gondii* exerting immunomodulation to inhibit breast cancer growth and metastasis. Parasit Vectors. 2021;14(1):601. 34895326 10.1186/s13071-021-05032-6 PMC8665513

[160] Darani HY, et al. Effects of *Toxoplasma gondii* and Toxocara canis antigens on WEHI-164 fibrosarcoma growth in a mouse model. Kor J Parasitol. 2009;47(2):175. 10.3347/kjp.2009.47.2.175 PMC2688801 19488426

[161] Pyo K-H, et al. Suppressed CD31 expression in sarcoma-180 tumors after injection with *Toxoplasma gondii* lysate antigen in BALB/c mice. Kor J Parasitol. 2010;48(2):171. 10.3347/kjp.2010.48.2.171 PMC2892575 20585536

[162] Yu-Meng J, et al. Effects of exreted/secreted antigens of *Toxoplasma gondii* on CD4 + CD25+ Foxp3 + T cells and NK cells of melanoma-bearing mice. Zhongguo Xue Xi Chong Bing Fang Zhi Za Zhi. 2011;23(3):301. 22164498

[163] Boghozian R, et al. Identification of *Toxoplasma gondii*protein fractions induce immune response against melanoma in mice. Apmis. 2015;123(9):800–9. 26152792 10.1111/apm.12420

[164] Pyo K-H, et al. Prominent IL-12 production and tumor reduction in athymic nude mice after *Toxoplasma gondii* lysate antigen treatment. Kor J Parasitol. 2014;52(6):605. 10.3347/kjp.2014.52.6.605 PMC4277022 25548411

[165] Yu-Meng J, et al. Inhibition of *Toxoplasma gondii* excretory-secretory antigens on growth of murine Lewis lung carcinoma. Zhongguo Xue Xi Chong Bing Fang Zhi Za Zhi. 2019;31(4):400. 31612675 10.16250/j.32.1374.2018269

[166] Seo S-H, et al. *Toxoplasma* GRA16 inhibits nf-κb activation through pp2a-b55 upregulation in non-small-cell lung carcinoma cells. Int J Mol Sci. 2020;21(18):6642. 32927892 10.3390/ijms21186642 PMC7554801

[167] Suzuki Y, Muto M, Kobayashi A. Antitumor effect of formalin-fixed Toxoplasma gondii organisms on EL4 lymphoma in *Toxoplasma*-infected mice. J Biol Response Mod. 1986;5(4):288–93. 3734844

[168] Yang MP, Ono GR, Suzuki K, Hasegawa N. Effect of *Toxoplasma* lysate antigen (TLA) on feline cytotoxicity against FeLV positive lymphoma cells. Jpn J Vet Sci. 1990;52(4):735–42. 10.1292/jvms1939.52.735 2167995

[169] Lu J, et al. Exosomes derived from dendritic cells infected with Toxoplasma gondii show antitumoral activity in a mouse model of colorectal cancer. Front oncol. 2022;12:p899737. 10.3389/fonc.2022.899737 PMC9114749 35600363

[170] Zhu S, et al. Anti-tumoral effect and action mechanism of exosomes derived from Toxoplasma gondii-infected dendritic cells in mice colorectal cancer. Front oncol. 2022;12:p870528. 10.3389/fonc.2022.870528 PMC9118538 35600340

[171] Amari A, et al. Effects of dendritic cell vaccine activated with protein components of *Toxoplasma gondii* on tumor specific CD8 + T-cells. Tehran Univ Med J. 2009;67(9).

[172] Motamedi M, et al. Improvement of a dendritic cell-based therapeutic cancer vaccine with components of *Toxoplasma gondii*. Clin Vaccine Immunol. 2009;16(10):1393–8. 19656994 10.1128/CVI.00199-09 PMC2756855

[173] Payne SN, et al. Novel murine pancreatic tumor model demonstrates immunotherapeutic control of tumor progression by a *Toxoplasma gondii* protein. Infect Immun. 2021;89(12). 10.1128/IAI.00508-21 PMC8594601 34543124

[174] Song Y, et al. The opposing effect of acute and chronic *Toxoplasma gondii* infection on tumor development. Parasit Vectors. 2024;17(1):247. 38835064 10.1186/s13071-024-06240-6 PMC11149184

[175] El Skhawy N, Shehata A, Eissa MM. Parasites’ immunomodulators: a breakthrough in immunotherapeutics displaying antineoplastic activity against human colorectal and hepatocellular carcinoma cells. Infect Agents Cancer. 2025. 10.1186/s13027-025-00715-6 PMC12805742 41387891

[176] Li J, et al. *Toxoplasma gondii* as a drug for anti-tumor immunotherapy: mechanisms, challenges, and perspectives. Parasite. 2026;33:4. 41641877 10.1051/parasite/2026006 PMC12875063

